# Heme-Oxygenases during Erythropoiesis in K562 and Human Bone Marrow Cells

**DOI:** 10.1371/journal.pone.0021358

**Published:** 2011-07-13

**Authors:** Liliane R. Alves, Elaine S. Costa, Marcos H. F. Sorgine, Maria Clara L. Nascimento-Silva, Cristina Teodosio, Paloma Bárcena, Hugo C. Castro-Faria-Neto, Patrícia T. Bozza, Alberto Orfao, Pedro L. Oliveira, Clarissa M. Maya-Monteiro

**Affiliations:** 1 Instituto de Bioquímica Médica, Universidade Federal do Rio de Janeiro, Rio de Janeiro, Brazil; 2 Instituto de Pediatria e Puericultura Martagão Gesteira, Universidade Federal do Rio de Janeiro, Rio de Janeiro, Brazil; 3 Centro de Investigación del Cáncer, Departamento de Medicina and Servicio de Citometria, Universidad de Salamanca, Salamanca, Spain; 4 Laboratório de Imunofarmacologia, Instituto Oswaldo Cruz, Fundação Oswaldo Cruz, Rio de Janeiro, Brazil; 5 Instituto Nacional de Ciência e Tecnologia em Entomologia Molecular, Rio de Janeiro, Brazil; Universidade de Sao Paulo, Brazil

## Abstract

In mammalian cells, heme can be degraded by heme-oxygenases (HO). Heme-oxygenase 1 (HO-1) is known to be the heme inducible isoform, whereas heme-oxygenase 2 (HO-2) is the constitutive enzyme. Here we investigated the presence of HO during erythroid differentiation in human bone marrow erythroid precursors and K562 cells. HO-1 mRNA and protein expression levels were below limits of detection in K562 cells. Moreover, heme was unable to induce HO-1, at the protein and mRNA profiles. Surprisingly, HO-2 expression was inhibited upon incubation with heme. To evaluate the physiological relevance of these findings, we analyzed HO expression during normal erythropoiesis in human bone marrow. Erythroid precursors were characterized by lack of significant expression of HO-1 and by progressive reduction of HO-2 during differentiation. FLVCR expression, a recently described heme exporter found in erythroid precursors, was also analyzed. Interestingly, the disruption in the HO detoxification system was accompanied by a transient induction of FLVCR. It will be interesting to verify if the inhibition of HO expression, that we found, is preventing a futile cycle of concomitant heme synthesis and catabolism. We believe that a significant feature of erythropoiesis could be the replacement of heme breakdown by heme exportation, as a mechanism to prevent heme toxicity.

## Introduction

Hematopoiesis is a tightly coordinated process involving a network of multiple signals, such as cytokines, hormones, and cellular cooperation, to promote hematopoietic stem cell (HSC) self-renewal and multilineage differentiation into distinct blood cell types. In adult mammals, bone marrow (BM) is the primary site for production of red blood cells (RBCs). The production of RBC requires an exceptionally high rate of heme biosynthesis to allow massive accumulation of hemoglobin in erythroblasts [1], [2]. It is well established in literature that heme acts as a regulator of its own synthesis, as well as a positive modulator of globin polypeptide chain synthesis in erythroblasts [3], [4]. Recently, heme has been ascribed a broader role as a critical modulator of cell signaling pathways, controlling gene expression, protein translation and post-translational protein modifications such as phosphorylation and ubiquitinylation [5], [6]. Based on the previous findings, heme-oxygenase has emerged, capable of controlling heme levels, preventing neuronal damage, regulating inflammation, and participating in vascular function [7], [8], [9].

Although heme is a signaling molecule and has obvious physiological relevance as the prosthetic group of hemeproteins, it is also a potential hazardous molecule capable of inducing reactive oxygen species leading to cell damage [10], [11]. Therefore, cellular heme levels are tightly regulated by a fine balance between its biosynthesis and catabolism [12]. Heme oxygenases are ubiquitous enzymes that degrade heme to ferrous iron, carbon monoxide (CO) and biliverdin. HO-2 isoform is constitutively expressed, whereas HO-1 is induced by multiple stress stimuli [13], [14], [15]. The primary mechanism to prevent free heme accumulation inside cells involves heme inducing HO-1 expression. According to the literature, it is generally assumed that intracellular free heme is rapidly degraded by HO, which is most frequently regarded as the major cytoprotective enzyme [16], [17]. In most cell types, induction of HO is accompanied by suppression of the ubiquitous isoform of 5-aminolevulinate synthase (ALAS-1), the rate-limiting reaction in the heme synthesis pathway [5]. The ALAS-2 isoform is exclusive to the erythroid lineage, and its expression is induced even in the presence of high heme concentrations during erythropoiesis [18], [19], [20]. Although increased availability of heme is known to be essential during red cell production, the regulation of the heme degradation pathway during erythroid differentiation has been largely overlooked.

In the study presented, we assessed the expression of HO isoforms during human erythroid differentiation. Initially, the *in vitro* model used a human erythroleukemia cell line (K562) undergoing heme-induced erythroid differentiation. The physiological pattern of HO expression during erythropoiesis was investigated in human bone marrow samples. The evaluation of heme metabolism enzymes in the different maturation stages of erythroid precursors was performed using multiparameter flow cytometry immunophenotyping. In addition, the presence of alternative routes to heme detoxification, such as the recently discovered heme exporter FLVCR (feline leukemia virus, subgroup C, receptor), was investigated.

## Materials and Methods

### Ethics Statement

It was used in this work samples obtained from human bone marrow. All subjects gave their informed consent prior to entering the study, and the study was approved by the local Ethics Committee of the University Hospital of Salamanca (Salamanca, Spain).

### Cell lines

The K562, THP-1 and RAW264.7 cell lines were obtained from the American Type Culture Collection (ATCC- Rockville, MD, USA). Murine RAW 264.7 cells were maintained in DMEM medium with 10% fetal bovine serum (FBS) (Invitrogen, Carlsbad, CA, USA) whereas both K562 and THP-1 human cell lines were cultured in RPMI 1640 (Invitrogen, Carlsbad, CA, USA), supplemented with 10% FBS. Cultures were maintained at 37°C in a 5% CO_2_ humidified incubator. In order to induce erythroid differentiation of K562 cells or analyze heme metabolism in two additional cell lines, cells were incubated with heme (0.2, 2.0 or 20 µM) for 24, 48, and 72 h. A 20 mM heme stock solution in dimethylsulfoxide (Me_2_SO) was diluted to 2 mM in 0.25 M NaOH. This solution was further diluted to 0.4 mM in culture medium immediately prior to use and was added to cell cultures at a desired final concentration. This serial dilution was used to preserve heme solubility in the final incubation media (pH 7.4).

### Patients and samples

A total of 10 ethylenediaminetetraacetic acid (EDTA)-anticoagulated BM samples were obtained from the same number of volunteers at the University Hospital of Salamanca (Salamanca, Spain) and analyzed in the present study. Within this group, six corresponded to BM aspirate samples obtained from lymphoma staging in patients which did not show evidence of any bone marrow expanded clonal population. One sample was from a woman that had two previous episodes of anaphylaxis and the absence of any clonal mast cell disease was found from BM analysis. In the other three BM aspirate samples, the diagnosis of myelodisplasic syndrome was excluded using both WHO criteria and the multiparametric flow citometry analysis. We performed the morphological and immunophenotypical evaluation of erythroid maturation in these BM samples, and all of them systematically showed patterns consistent with normal differentiation. All subjects gave their informed consent prior to entering the study, and the study was approved by the local Ethics Committee of the University Hospital of Salamanca (Salamanca, Spain).

### Western Blot studies

Cell lysates were prepared using SDS-sample buffer containing β-mercaptoethanol; they were submitted to electrophoresis in 7.5–20% acrylamide gradient SDS-PAGE gels [21]. All the westerns were performed with the usage of the Prestained Rainbow Molecular Weight Marker (GE Healthcare). The samples were transferred onto nitrocellulose membranes (Bio-Rad, Hercules, CA, USA) and non-specific binding sites were blocked with 5% non-fat milk in Tris buffered saline-Tween 20 (TBST; 50 mM Tris-HCl, pH 7.4, 150 mM NaCl, 0.05% Tween 20). Membranes were probed with anti-HO-1, anti-HO-2 (Assay Designs, Ann Arbor, MI, USA) and anti-β-actin (BD Biosciences, Franklin Lakes, NJ, USA) monoclonal antibodies (mAb) in TBST with 1% non-fat dry milk. The membranes were incubated with a horseradish peroxidase (HRP)-conjugated secondary antibody in TBST, followed by detection of antigen-antibody complexes using a Supersignal Chemiluminescence kit (Pierce, Rockford, IL, USA). Densitometric analysis of images from developed film was performed using the Image 2D software (GE Healthcare).

### Real-time PCR analysis

Whole RNA samples derived from the three cell lines were extracted from 10^6^ cells using TRIzol reagent (Invitrogen, Carlsbad, CA, USA). RNA extraction from human BM cells was performed using the Nucleospin RNA XS Kit (Macherey-Nagel, Düren, Germany), as recommended by the manufacturer. First strand cDNA was synthesized using the *High Capacity cDNA Reverse Transcription Kit* (Applied Biosystems, Foster City, CA, USA), following the specification provided in the kit protocol. PCR amplification of the selected genes was carried out using specific primers, as described in Table S1. Real-Time PCR was performed in an ABI 7500 Real-Time PCR System (Applied Biosystems) using the SYBR Green I double-stranded DNA-specific fluorophore (Power SYBR® Green PCR Master Mix; Applied Biosystems). cDNAs were amplified using a 40-cycle reaction of denaturation (15 s, 95°C) and annealing/extension (60 s, 60°C). Formation of a single product was determined by melt curve analysis. Quantification of mRNA was performed by comparative Ct and GAPDH mRNA was used as endogenous reference. The fold increase above control levels was obtained through the calculation of 2 −ΔΔCt, where ΔΔCt = ΔCt treatment−ΔCt control [22].

### Multiparameter Flow Cytometry immunophenotyping studies

In order to evaluate HO-1 expression, whole BM samples were fixed with solution A from the Fix & Perm™ kit (Invitrogen) for 15 min at room temperature (RT); they were subsequently washed with PBS containing 0.2% bovine serum albumin (BSA) and centrifuged for 5 min at 540 g. The BM cells were incubated (15 min at RT) with solution B from the Fix & Perm™ and FITC-conjugated anti-human HO-1 mAb (Assay Designs, Ann Arbor, MI, USA) and washed with PBS containing 0.2% BSA (5 min at 540 g). Assessment of FLVCR expression was performed on erythrocyte-lysed BM samples in FACS-lysing solution [Becton Dickinson Biosciences (BD), San José, CA] diluted 1∶10 (v/v) in distilled water. Briefly, erytrocyte-lysed samples were sequentially washed in PBS containing 0.2% BSA, incubated with an anti-FLVCR mAb (ABNOVA) for 15 min (RT) and washed in PBS containing 0.2% BSA. Next, the cells were incubated with a FITC-conjugate anti-mouse IgG reagent (Cytognos SL, Salamanca, Spain) for 15 min in the dark (RT) and were washed in PBS containing 0.2% BSA. Finally, samples stained with HO-1 and FLVCR were both incubated (15 min at RT in the darkness) with two different combinations of fluorochrome labeled MAb reagents. These were used to define the distinct stages of maturation of BM erythroid precursors (CD45-Pacific Blue, CD71-APC, CD105-PE and CD34-PerCP-Cy5.5) and to specifically identify BM erythroid precursors and monocytes (CD45-Pacific Blue, CD71-APC, CD36-PE). A final wash step was performed with PBS (5 min at 540 g).

Stained BM samples were analyzed in a FACS Canto II flow cytometer equipped with FACSDiVa software (BD); data analysis was performed with the Infinicyt software (Cytognos). BM erythroid precursors were identified as FSC^lo^/SSC^lo^/CD34^−^/CD45^−^/CD71^++^/CD61^−^ cells, and monocytes as FSC^hi^/SSC^lo^/CD34^−^/CD36^+^/CD45^hi^/CD61^−^ cells (figure S1). Purification of different compartments of stained BM cells was performed with a FACSAria flow cytometer (BD), as illustrated in Figure S1. FACS-sorted cells were collected in PBS and re-analyzed for purity, which was systematically >98%. Sorted cells were stored at −70°C in solution RA1 of the Nucleospin RNA XS Kit (Macherey-Nagel).

### Statistical Analyses

Data were reported as mean values ± SEM and were analyzed statistically by means of analysis of variance followed by a Student t-test using the SPSS software package (SPSS, Chicago, IL, USA) or by the one-way analysis of variance and a posteriori Tukey's test, using GraphPad Prism version 5.00 software (GraphPad Software, San Diego, CA, USA).

## Results

### Induction of synthesis of hemoglobin by heme in K562 cells

**Figure 1:**
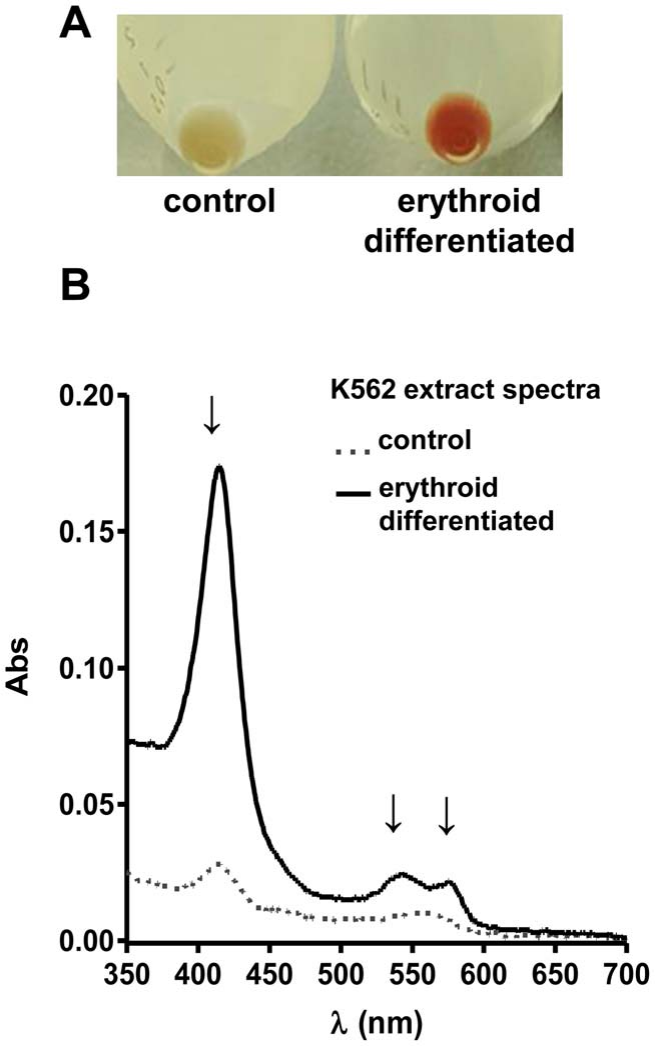
Heme induces erythroid differentiation by increasing hemoglobin synthesis in K562 cells. K562 cells were cultured for 48 h in the absence or presence of heme (20 µM) (A) cell pellet of erythroid differentiated culture with an intense red color when compared with the control. (B) Spectrophotometric analysis of the control (**^…..^**) and heme stimulated (**^___^**) cell lysates obtained from the pellets shown in panel A. Visible light absorption spectrum characteristic of hemoglobin. The arrows indicate the characteristic wavelenght peaks of hemoglobin absorption the Soret band at 415 nm and α and β bands at 542 nm and 577 nm.

The human K562 erythroleukemia cell line mimics a common megakaryocyte-erythroid BM progenitor [23]. K562 cells have been used as a model for erythropoiesis since exposure of these cells to heme and other substances, such as sodium butyrate, have been reported to increase expression of globin mRNA [18], [24], [25]. As heme is likely a physiological stimulus, heme-induced erythroid differentiation under cell culture conditions was studied. Incubation of K562 cells with heme (20 µM) for 48 h strongly induced hemoglobin synthesis, as evidenced by the intense red color of the cell pellet (Figure 1A). The color is a consequence of the light absorption spectrum characteristic of hemoglobin with the heme bound at heme-pocket (Soret band at 415 nm and α and β bands at 542 nm and 577 nm) (Figure 1B) [26].

### Absence of heme-oxygenase 1 in K562 cells

**Figure 2:**
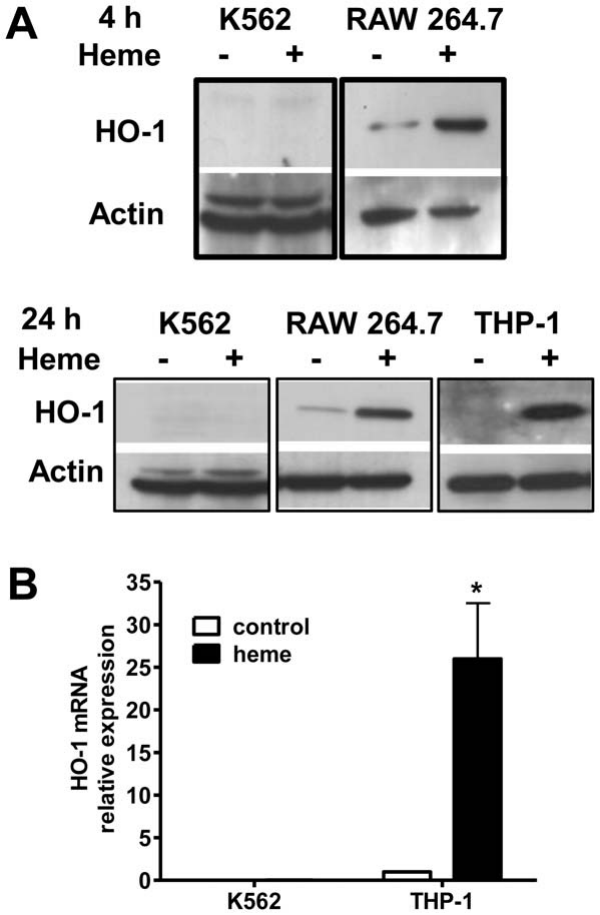
Modulation of heme-oxygenase 1 by heme during erythroid differentiation of K562 cells. (A) Western Blot analysis of K562 and RAW 264.7 cell lysates for HO-1 expression. K562 and RAW 264.7 cultures were incubated with or without heme (20 µM) for 24 h. RAW 264.7 was a positive control of heme oxygenase-1 induction after 4 h or 24 h of heme treatment. Actin detection serves as a loading control. (B) Relative expression of HO-1 mRNA in K562 and THP-1 cells under control conditions and after stimulation with heme (20 µM) for 24 h. The graph displays the average values of three independent experiments with internal biological triplicates of a real-time PCR quantification assays. Results were systematically normalized by GAPDH expression. The THP-1 control was used as a reference to calculate the relative expression, and it was set at one. The analysis of variance (ANOVA) indicated significant differences (*p<0.001) for THP-1 but not K562 cells.

Heme is known to increase expression of HO-1, the inducible isoform of HO. Therefore, K562 was used as a model to analyze the heme degradation pathway under a condition of intense hemoglobin synthesis. When incubated only with culture medium, no detectable expression of HO-1 in K562 cells was found as observed by western blot and confirmed by real-time PCR (Figure 2 A, B). Moreover, exposure of K562 cells to high concentrations of heme (20 µM) did not elicit HO-1 expression after 4 h or 24 h (Figure 2A). The lack of measurable expression in K562 cells was also observed at different time points after addition of heme to the culture medium (48 and 72 h; data not shown). RAW264.7 cells (mouse macrophage cells) and THP-1 human monocytic cells were used as positive controls, as incubation in the presence of heme promptly (4 h and 24 h) resulted in strong expression of HO-1 (Figures 2A and B).

### Heme downregulation of heme-oxygenase-2 expression

**Figure 3:**
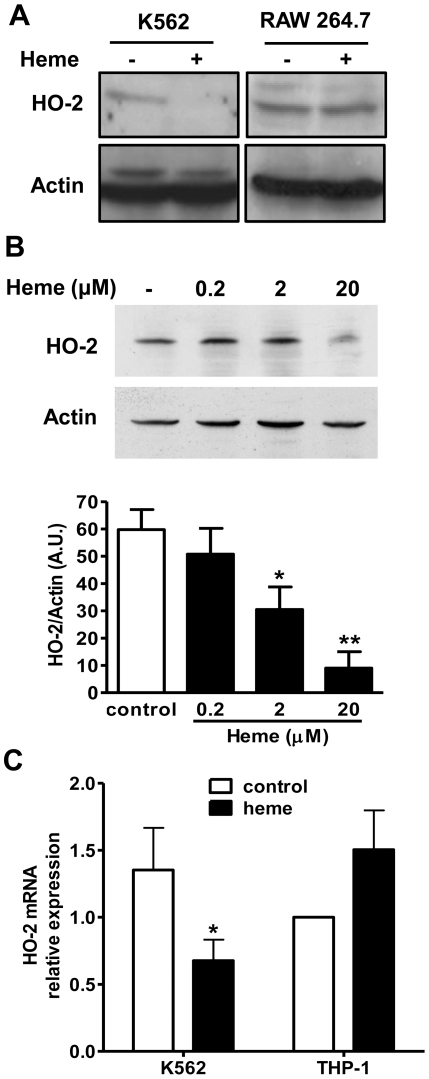
Heme inhibits the constitutive isoform of heme oxygenase (HO) HO-2 in K562 cells. (A) Western Blot analysis of K562 and RAW 264.7 cell lysates to determine HO-2 expression. K562 and RAW 264.7 cell cultures were incubated in absence or presence of heme (20 µM) for 48 h. Actin detection was used as loading control. (B) Western Blot analysis of K562 cells cultured with increasing concentrations (0, 0.2, 2.0 or 20 µM) of heme. The graph represents the results of three independent experiments normalized by actin expression. (C) Relative expression of HO-2 mRNA in K562 and THP-1 cells incubated in the presence or absence of heme (20 µM) for a period of 24 h. The graph shows the average results of three independent experiments with internal biological triplicates of a real-time PCR assay, normalized by GAPDH expression. The THP-1 control was used as a reference to calculate relative expression, and it was set at one. * p<0.05; ** p<0.01.

Heme-oxygenase 2 is usually referred as the constitutively expressed isoform of HO and is usually not explored in most studies addressing regulation of heme degradation. As HO-1 was not induced in K562 cells, a possible compensatory stimulation of HO-2 was investigated. Unexpectedly, instead of increased HO-2 expression, exposure of K562 cells to heme inhibited HO-2 protein expression in a dose-dependent mode (Figure 3A and 3B). As expected in RAW 264.7 cells, the HO-2 levels were not influenced by heme (Figure 3A). The results obtained by Western blot were further supported by real-time PCR, which indicated that HO-2 mRNA was inhibited under heme-induced erythroid differentiation (Figure 3C). As expected, the human control THP-1 cells showed a lack of modulation of HO-2 by heme.

### Lack of heme oxygenase-1 and inhibition of HO-2 expression in human bone marrow erythroid precursors

**Figure 4:**
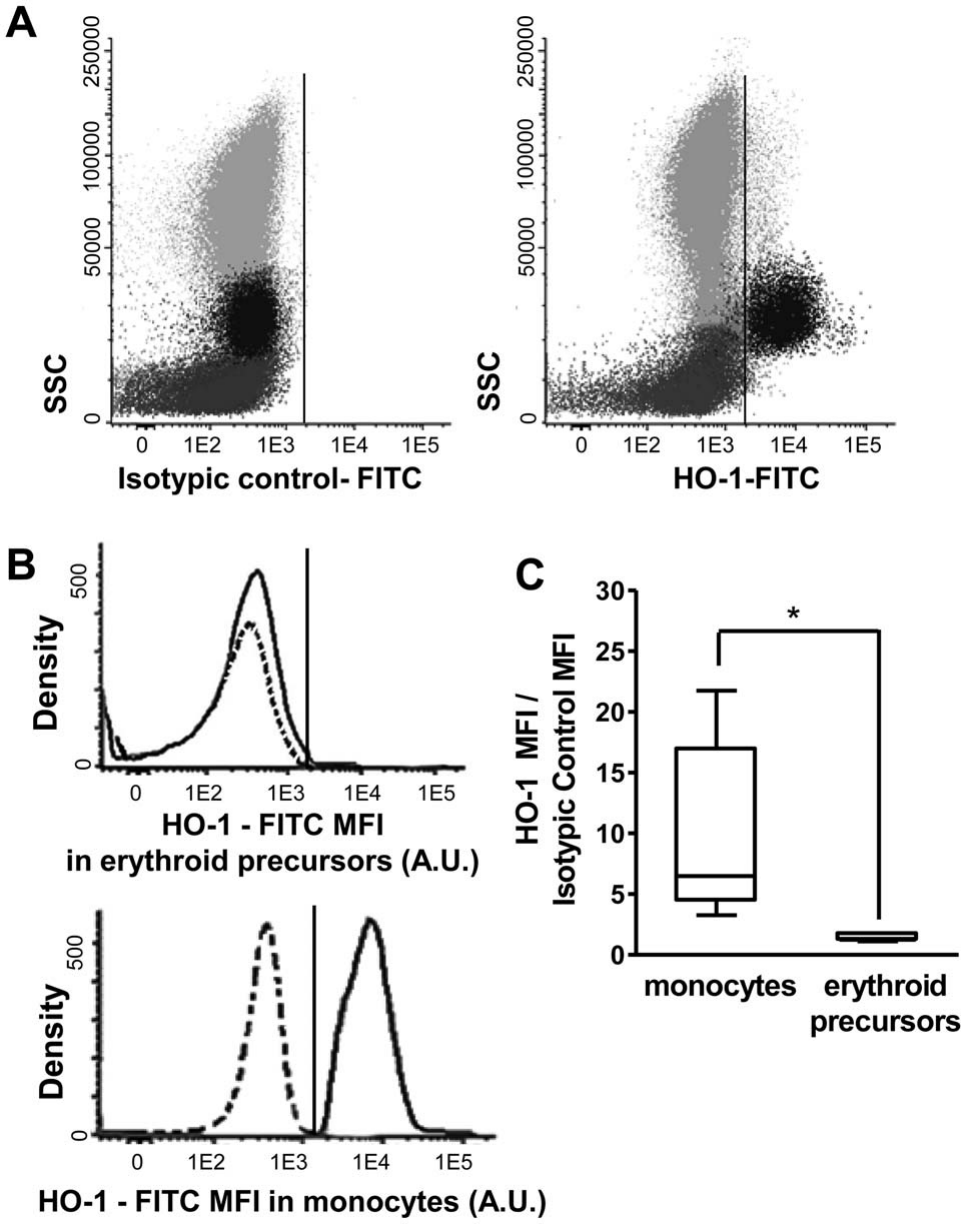
Expression of HO-1 by normal human BM erythroid precursors and monocytes. (A) Flow cytometry analysis of HO-1 antigen expression in erythroid precursors (dark grey dots) gated as FSC^lo^/SSC^lo^/CD34^−^/CD45^−^/CD71^++^/CD61^−^ cells and the monocytic population (black dots) were identified as FSC^hi^/SSC^lo^/CD34^−^/CD36^+^/CD45^hi^/CD61^−^. Other BM cells are displayed as light gray events/dots. Expression of HO-1 (A right panel) was compared to the isotypic control (A left panel). (B) Histogram obtained from the date in panel A comparing the fluorescence intensity of isotypic control (dotted line) and anti HO antibody (continuous line) in erythroblasts or monocytes. (C) Box plot representing mean fluorescence intensity (MFI, arbitrary units – A.U.-form 0 to 262144 channels) values obtained for HO-1 protein expression divided by the MFI of the isotypic control for normal human BM erythroid precursors and monocytes in five bone marrow samples described under Methods (*p = 0.01).

The unusual behavior of the heme degradation pathway could be attributed to an anomaly unique to K562 malignant cell line. Investigations were performed to determine if the down-regulation of HO-2 and the lack of HO-1 expression would also be found during normal erythropoiesis in human BM. Erythroid precursors from BM samples showed undetectable levels of HO-1 expression, similar to what was found in K562 cells (Figure 4A and Figure 4B). These results suggested that in human BM, normal erythropoiesis occured without heme degradation by HO-1. Noteworthy, HO-1 expression was clearly limited to the monocyte population while the other BM cell compartments showed an absence of labeling for HO-1 (Figure 4A and Figure 4B).

**Figure 5:**
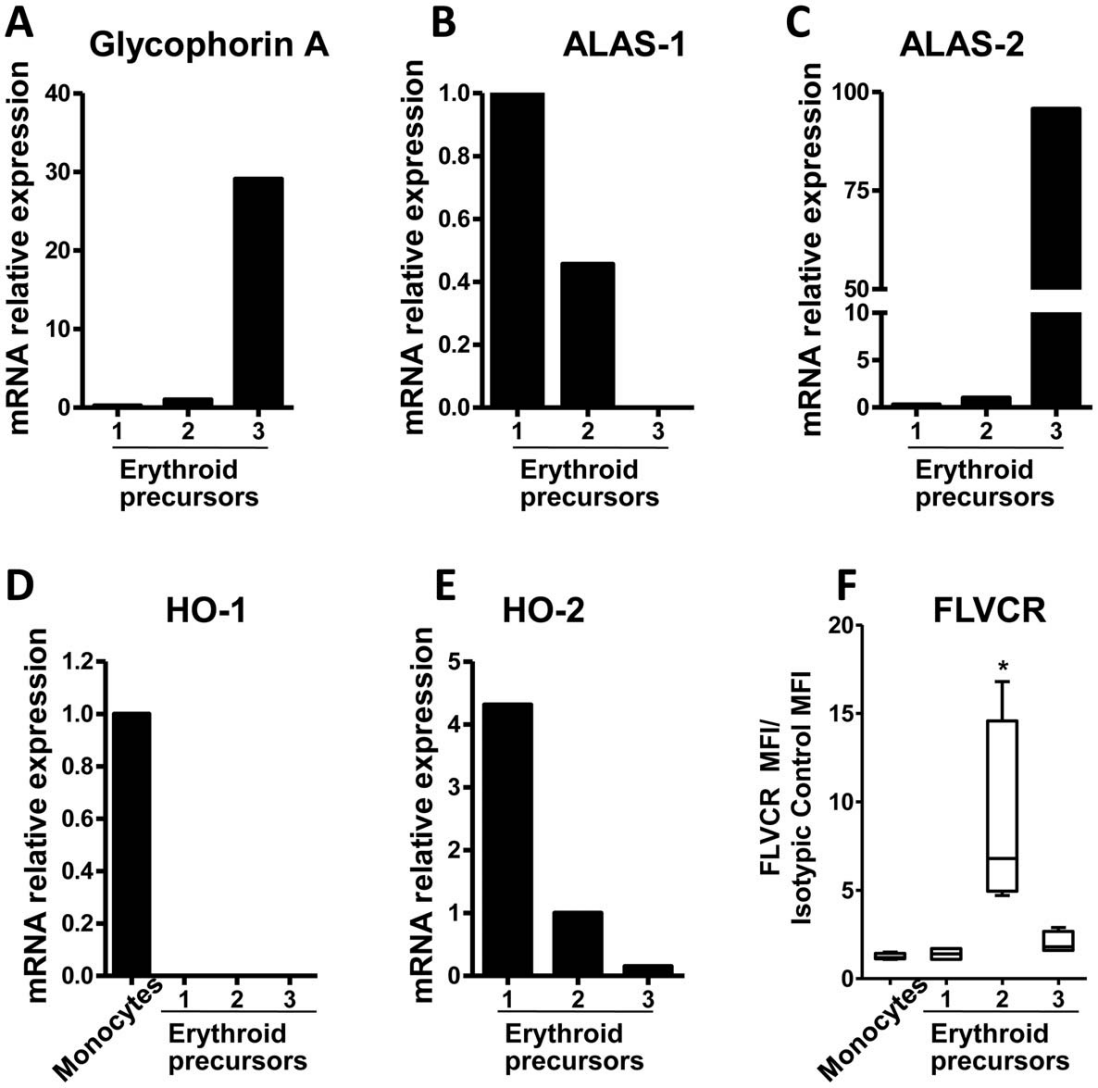
Profile of heme metabolic enzymes, FLVCR and glycophorin A expression during erythroid maturation in adult human BM. (A–E) Expression levels of mRNA for glycophorin A, ALAS-1, ALAS-2, HO-1 and HO-2 at different stages of maturation of erythroid precursors (**stage 1:** CD45^−^, CD105^+^, CD71^+^ cells; **stage 2:** CD45^−^, CD105^++^, CD71^+^ cells, and **stage 3:** CD45^−^, CD105^−^, CD71^++^ cells) and **monocytes** (in D) (CD45^+^, CD36^+^, CD34^−^ cells) as detected by Real Time PCR. The histograms are representative of five independent experiments using BM samples from five patients described under Methods, with internal triplicates, normalized by GAPDH expression on FACS-purified populations (see Figure S1). (F) Flow cytometric expression of FLVCR is shown for the same cell subpopulations. The box extends from the 25^th^ to the 75^th^ percentile and the line at the middle is the median. The whiskers extend down to the lowest value and up to the highest. Analysis of variance (ANOVA) and Tukey's Test was made to indicate statistical differences (*p<0.01).

In order to gain insight into the regulation of heme catabolism during erythropoiesis, human BM erythroid precursors were further classified into three maturation-associated subpopulations according to the pattern of expression of both CD105 and CD71(Figure S1) [27], [28]. The more immature erythroid precursors showed a CD105^−^/CD71^lo^ phenotype, intermediate cells were CD105^+^/CD71^hi^, and the major compartment of more mature nucleated red blood cells (NRBC) showed a CD105^−^/CD71^hi^ phenotypic profile (Figure S1). The maturation of these three BM NRBC compartments was validated by real-time PCR, which showed a progressive increase in mRNA expression of both glycophorin A and ALAS-2, that are respectively, a major component of the red cell membrane and the erythroid-specific isoform of ALAS (Figure 5A and C). The progression of erythroid maturation in the subpopulations that were identified using this approach received further support from the decrease in the expression of ALAS-1, which was known to occur during erythropoiesis (Figure 5 B) [29], [30], [31]. BM monocytes were used as internal positive control for HO-1 (Figure 5 D).

Similar to what was described above (Figure 4), real-time PCR revealed that HO-1 mRNA levels were actually below detection limits in all BM compartments of NRBC precursors (Figure 5D). Remarkably, HO-2 mRNA expression was progressively down-regulated during the course of erythroid differentiation (Figure 5E).

### FLVCR expression during normal human erythropoiesis

**Figure 6:**
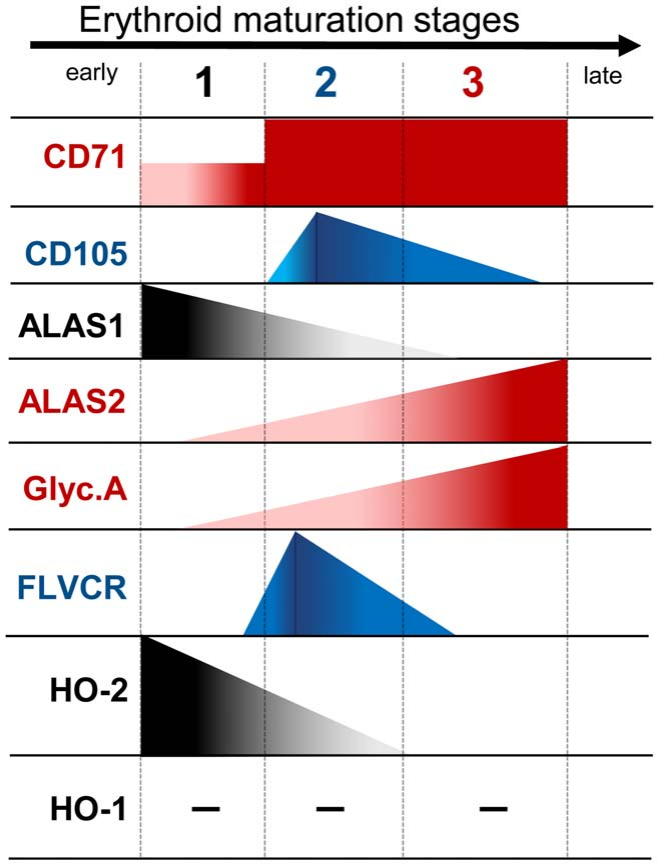
Schematic representation of heme oxygenase (HO-1 and HO-2) and FLCVR expression during human BM erythropoiesis. Expression profile of FLVCR, HO-1 and HO-2 and ALAS-1, ALAS-2 and Glycophorin A (Glyc.A) is displayed at different stages of BM erythroid precursors that are defined according to the expression of CD71 and CD105.

Erythropoiesis was characterized by a dramatic increase in the intracellular heme/hemoglobin contents. Since erythroid precursor cells lack HO-1 and there is a progressive decrease in HO-2 expression during maturation, we hypothesized that these cells might use alternative mechanisms to protect themselves against heme overload. Recently, Keel et al. [32] reported that the FLVCR heme exporter plays an important role during erythroid differentiation. Analysis of FLVCR expression during normal BM maturation of erythroid precursors showed that maximal expression occurs at the intermediate (CD105^+^/CD71^hi^) stage of maturation (Figure 5F). In Figure 6 it is shown the erythroid differentiation protein expression pattern, considering our data for the human bone marrow on figure 4, figure 5 and figure S1.

## Discussion

Commitment of hematopoietic stem cells to the erythroid lineage was accompanied by expression of proteins such as glycophorin A, ALAS-2 and by intense production of hemoglobin and heme. Reorganization of the heme and iron metabolism in a unique manner is a key step in the making of the red cell [33]. The ALAS-1 isoform is inhibited by a high intracellular concentration of heme, while the ALAS-2 is not repressed by heme; in turn, ALAS-2 is responsible for intense heme synthesis during erythropoiesis and the increased availability of heme is required for hemoglobin synthesis [18], [19], [34]. Intracellular heme is able to stimulate globin mRNA translation. There are known redundant pathways to modulate globin and heme synthesis in those cells [20], [35], [36]. Comparatively, little is known about the role of HO enzymes during the formation of red cells. Elevated concentrations of heme are not found in most non erythroid cells. HOs promptly degrades free heme from *de novo* synthesis that is not incorporated into hemeproteins, or from degradation of senescent hemeproteins during protein turnover. It is shown here that the need to build-up high levels of intracellular heme during erythropoiesis is accompanied by suppression of heme degradation by heme-oxygenases.

HO-1 is the inducible isoform of heme-oxygenase, whose expression is strongly stimulated by heme. This is true for the majority of the mammalian cells studied thus far. K562 cells have been extensively used as a model to study erythroid differentiation. Although some reports in the literature showed a heme degradation pathway in K562 [18], [37], we were not able to detect HO-1 expression in K562 cells, even when exposed to high concentrations of heme. Some authors used K562 cells transfected with constructs expressing the HO-1 gene, instead of studying native expression of HO-1 [38]. This could be attributed to the fact that most of those previous reports have evaluated heme-oxygenase enzyme activity without identifying the isoform involved [39]. Lavrovsky et al. [40] has identified HO-1 expression in K562 based on Northern blot experiments. These discrepancies could be attributed to the use of more sensitive and specific methods (Q-PCR and western blot), different from those employed in the previous reports. We concluded that the results presented indicate that the previous reports on HO activity in K562 cells may reflect the presence of the HO-2 isoform and not HO-1.

The results obtained with the K562 cell line were intriguing and led us to investigate the pattern of HO-1 expression under physiological conditions in human bone marrow. In support of these findings, human BM erythroid precursors at different stages of maturation also showed undetectable amounts of both the HO-1 protein and mRNA. Macrophages are responsible for degradation of most of the heme from hemoglobin in mammalian organisms, and are known to express HO-1 [15], [41], [42]. Coherently, high levels of HO-1 were found both in the THP1 and RAW cell lines, as well as in human BM monocytic precursors, serving as a positive control for heme-induced HO-1 expression. Because monocytes have a common precursor with the erythroid lineage, the results suggest that loss of HO-1 expression is an early event during erythropoiesis, which could even occur at the split between the monocyte/neutrophil *vs* red cell/platelet committed precursors.

Although HO-2 is known to be a constitutive enzyme that is frequently ascribed a housekeeping role, reports have shown that its expression is actively modulated in response to ischemia/hypoxia [43] or tissue injury [44]. This led us to evaluate a possible role of HO-2 in compensating for the loss of HO-1 in the erythroid precursors. Our results indicated the opposite, a marked down-regulation of expression of HO-2 during human bone marrow erythroid differentiation. We do not know the correct mechanism that is responsible for the reduction of HO-2 activity under physiological conditions. However, in K562 cells, HO-2 expression was down-regulated in response to exposure to increased levels of heme. That suggested that increased heme may be the signaling event that, in the erythroid precursors, is responsible for suppression of heme degradation.

A question that emerged from these results was why in the erythroid precursor lineage heme should prevent its own degradation, in opposition of what has been described for all other cell types. Most literature reports pointed to an anti-oxidant property of HO because heme is known to be a pro-oxidant molecule. However, it has been shown that cleavage of heme by HO can also exert a pro-oxidant effect under specific conditions; this is particularly true when an increased iron availability exists, since the reaction catalyzed by HO generates free iron that can participate in the Fenton reaction [10]. During erythropoiesis, it has been shown that ferritin synthesis is down-regulated to increase iron availability and heme biosynthesis and allows for the accumulation of hemoglobin [34]. The reduced HO activity reported could prevent both iron overload and occurrence of oxidative stress. Also, HO suppression may prevent an unnecessary cycle of concomitant heme synthesis and catabolism. Reduction of total HO activity during erythropoiesis has been previously reported. Hoffman et al [45] and Trakshel et al [46] have shown that heme reduced HO total activity in K562 cells, and Fujita et al [47] reported that MEL cells (Friend-virus transformed erythroleukemia) exhibited a transient alteration of HO activity. However, these pioneering observations did not receive attention in the literature, probably because HO isoforms themselves had not been discriminated and have only recently been recognized to play a major regulatory role in cell physiology.

The hypothesis that disruption of the heme degradation pathways could reflect an adaptation to allow for hemoglobin accumulation during erythropoiesis led us to search for additional mechanisms that could also contribute to prevent or counteract heme toxicity. Recently, Quigley [48] showed that the Feline Leukemia Virus Cell Receptor acts as a heme exporter protein which is essential to erythropoiesis. Resting K562 cells showed low expression of FLVCR; however, when these cells were committed to erythroid differentiation, inhibition of this heme exporter promotes apoptosis. Later, Keel et al. [32] demonstrated that disruption of FLVCR expression in mice promoted defective erythropoiesis and the animals died during gestation. Furthermore, mutations that produced abnormal spliced forms of FLVCR were associated to low expression of FLVCR in Blackfan-Diamond anemia, indicating that this may be relevant for the establishment of the disease [49]. It was proposed that these effects could be a consequence of incomplete erythropoiesis due to inability to export the excess of heme. It was discovered that FLVCR expression increased in human BM nucleated red blood cell precursors (NRBC) during the course of erythropoiesis, peaking at the intermediate stage of maturation, and decreasing at later stages in the more mature CD105^−^/CD71^hi^ NRBC (figure 6). This result is consistent with the key role suggested for FLVCR during erythropoiesis [32]. As these cells do not express HO-1 and show progressive suppression of HO-2 expression, FLVCR-mediated heme export might be understood as a safeguard mechanism that would prevent accumulation of heme in excess of the capacity of the cell to use it for hemoglobin synthesis.

In summary, the data indicated that the mechanisms of regulating heme degradation described to be common to eukaryotic cells are subverted during erythroid maturation, where suppression of HO expression plays a key role in cell differentiation.

## Supporting Information

Figure S1 **The sequential gating strategy followed to identify the cell populations corresponding to nucleated red blood cell (NRBC) precursors and monocytes in adult normal human BM (Panels A to D).** In panels E to M, gating of BM NRBC different maturation stages is illustrated from the more immature precursors (panels E to G) to NRBC at intermediate (panels H to J) and more advanced stages of maturation (panels K to M).
(DOC)

Table S1 **Oligonucleotides used in Real-Time PCR assays.** Oligonucleotide sequences used as primer foward/reverse to determine the relative expression of heme-oxygenase 1 (HO-1), heme-oxygenase 2 (HO-2), ALA-synthase 1 (ALAS-1), ALA-synthase 2 (ALAS-2), FLVCR, Glycophorin A and Glyceraldehyde-3-phosphate dehydrogenase (GAPDH) genes by Real-Time PCR.
(DOC)
