# Supplementary material for: Heme-Oxygenases during Erythropoiesis in K562 and Human Bone Marrow Cells
Source: PLoS One. 2011 Jul 13;6(7):e21358. doi: 10.1371/journal.pone.0021358 (PMC3135583; doi:10.1371/journal.pone.0021358)
Supplement: Table S1 — Oligonucleotides used in Real-Time PCR assays. Oligonucleotide sequences used as primer foward/reverse to determine the relative expression of heme-oxygenase 1 (HO-1), heme-oxygenase 2 (HO-2), ALA-synthase 1 (ALAS-1), ALA-synthase 2 (ALAS-2), FLVCR, Glycophorin A and Glyceraldehyde-3-phosphate dehydrogenase (GAPDH) genes by Real-Time PCR. (DOC) [file pone.0021358.s002.doc]

**Supplementary data**

Supplementary Table 1: Oligonucleotide sequences used in Real-Time PCR assays.

| **Gene** | **Primer foward/reverse sequences (5`- 3`)** |
| --- | --- |
| HO-1 | ATGACACCAAGGACCAGAGC/TCTCCGATGGGTCCTTACAC |
| HO-2 | CACGATGGGAAAGGAGACAT/CCCTAGCTGCTGGACTCTTG |
| ALAS-1 | CCTCCGGCCAGTGAGAAAGA/CTGCCAGGAAAGGGCATTTG |
| ALAS-2 | ACAGTGCTGCCCAGTGCTTG/TCCGACAGCATGAAGGGACA |
| FLVCR | TGACCATTCTCCTTTGAGGAA/CAGTGGTAGGCACTGTGGAA |
| Glycophorin A | atacgcacaaacgggacaca/taccctttctccggtttcct |
| GAPDH | CCTGTTCGACAGTCAGCCG/CGACCAAATCCGTTGACTCC |
