# Supplementary material for: Heme-Oxygenases during Erythropoiesis in K562 and Human Bone Marrow Cells
Source: PLoS One. 2011 Jul 13;6(7):e21358. doi: 10.1371/journal.pone.0021358 (PMC3135583; doi:10.1371/journal.pone.0021358)
Supplement: Supplementary file 2 [file pone.0021358.s001.doc]

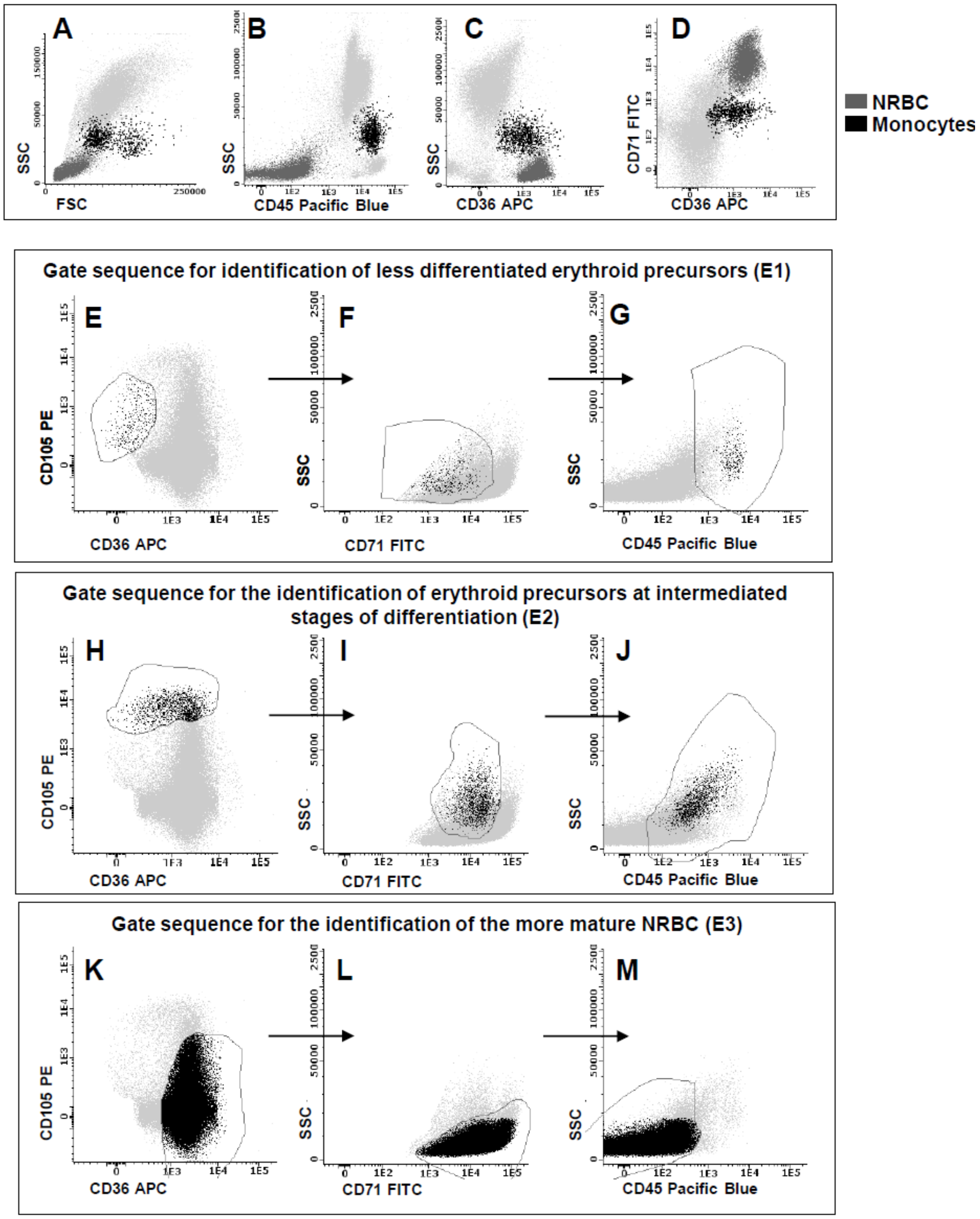
A
SSC
FSC
B
SSC
CD45 Pacific Blue
C
SSC
CD36 APC
D
CD71 FITC
CD36 APC
NRBC
Monocytes
Gate sequence for identification of less differentiated erythroid precursors (E1)
E
CD105 PE
CD36 APC
F
SSC
CD71 FITC
G
SSC
CD45 Pacific Blue
Gate sequence for the identification of erythroid precursors at intermediated stages of differentiation (E2)
H
CD105 PE
CD36 APC
I
SSC
CD71 FITC
J
SSC
CD45 Pacific Blue
Gate sequence for the identification of the more mature NRBC (E3)
K
CD105 PE
CD36 APC
L
SSC
CD71 FITC
M
SSC
CD45 Pacific Blue
